# Physical Activity and the Emotional State of Physiotherapy Students Who Finish Their Education

**DOI:** 10.3390/ijerph18094572

**Published:** 2021-04-26

**Authors:** Joanna Kowalska, Dorota Wójtowicz, Joanna Szczepańska-Gieracha

**Affiliations:** Department of Physiotherapy, University School of Physical Education, 51-612 Wroclaw, Poland; dorota.wojtowicz@awf.wroc.pl (D.W.); joanna.szczepanska@awf.wroc.pl (J.S.-G.)

**Keywords:** depressive symptoms, sense of coherence, stress, students, physiotherapy, physical activity

## Abstract

This study aimed to evaluate the emotional state and the level of the sense of coherence in the context of physical activity among physiotherapy students and investigate how the participating students’ emotional state changed after two years of studying and what factors were associated with the mood disorders. The study group consisted of 110 students—79 female and 31 male. The Back Depression Inventory (BDI), the Sense of Coherence Questionnaire (SOC-29) and the Perceived Stress Questionnaire (PSQ) were used. The results presented an increase in depressive symptoms during the studies of a group of physiotherapy students. Physical activity can play a protective role in the prevention and treatment of mood disorders. The students who regularly engaged in physical activity exhibited better mood, a lower perceived stress level and a higher level of the sense of coherence as compared to the physically inactive students.

## 1. Introduction

The existing research indicates that the incidence of depression among students rises as their studies progress [1,2]; in contrast, other publications demonstrate that the symptoms of depression are significantly more severe in first-year students [3]. Nonetheless, this phenomenon primarily affects the students of higher education institutions, majoring in medical fields, including physiotherapy, i.e., those preparing to enter the professions associated with helping other people [4,5,6].

Numerous stress factors, sleep deprivation, an unbalanced diet, the lack of regular physical activity, fatigue, high expectations, both their own and those of the society, an increased sense of responsibility while taking independent decisions and taking charge of their own lives, but primarily the contact with the suffering of other individuals and an insufficient support system increase the risk for the symptoms of depression developing [7,8].

The students majoring in those fields—despite the fact that they acquire the necessary knowledge on mental disorders during their studies—do not consider depression to be a condition that requires therapy [8]. Notably, the majority of those students do not seek help and they receive proper treatment less frequently than the members of the general population who suffer from depression [9]. Additionally, although they are aware of the protective function of physical activity in the prevention and treatment of multiple conditions, they engage in it less frequently [10,11,12,13]. These findings are very alarming due to the nature of the work that those students will perform in the future.

A protective role in the development of emotional disorders is also attributed to the sense of coherence [14]. In accordance with Antonovsky’s salutogenic model, this is a key factor for being able to successfully cope with multiple stressors and maintain good health, which is influenced by the processes of growth and development throughout the individual’s life based on the experiences they gain [15].

Therefore, it appears advisable (despite the existing, albeit not very extensive research) to regularly monitor the emotional state of the students majoring in medical fields, including physiotherapy, to identify the individuals who require help and determine the factors that accompany emotional disturbances. This is particularly important in the ever-changing world of needs, expectations, and demands that awaits young people who enter adulthood.

Therefore, the aim of the study was to evaluate the emotional state and the level of the sense of coherence in the context of physical activity among physiotherapy students and to investigate how the emotional state of the participating students changed after two years of studying and what factors were associated with the mood disorders in the group of students who were about to finish their education.

## 2. Materials and Methods

### 2.1. Participants and Procedure

The study was carried out between 2016 and 2019 at the Department of Physiotherapy of the University School of Physical Education in Wroclaw among the third-year bachelor’s degree students majoring in physiotherapy (initial study–T1), who were subsequently re-evaluated two years later as second-year Master’s degree students (final study–T2). The results of the initial study (N = 249) were presented in detail and discussed in an article published in WORK—A Journal of Prevention Assessment & Rehabilitation [13]. For this publication, only those participants who completed both the initial (T1) and the final (T2) questionnaire after two years of study were taken into account during the analysis. Ultimately, the study group consisted of 110 students—79 female and 31 male. All students consented to participate in the study and complete the questionnaires. The students were informed about the purpose of the study and the possibility to withdraw at any stage. The study was carried out in the form of a questionnaire, without any intervention or experiment structure, with the participants’ consent and under the ethical and legal supervision of the Department of Physiotherapy of the University School of Physical Education in Wroclaw and the study was conducted in accordance with the Helsinki Declaration.

The characteristics of the study group are presented in Table 1.

### 2.2. Measure Tools

The Beck Depression Inventory (BDI), the Sense of Coherence Questionnaire (SOC-29) and the Perceived Stress Questionnaire (PSQ) were used in the study. Additionally, the students answered other questions, e.g., about their physical activity; financial situation; place of residence; satisfaction with the chosen field of study; future plans after finishing education; friendships; and the need to consult a psychologist.

The BDI is a screening tool used for determining the presence of the symptoms of depression. It is a 21-item inventory consisting of two parts: emotional and somatic. The score of 0–11 points indicates no depression, 12–19—mild depression, 20–25—moderate depression and 26–63—severe depression. The BDI has very high internal consistency—Cronbach’s alpha for the entire standardization sample is 0.93 and for patients suffering from depression—0.95 [16].

The SOC-29 comprises 29 questions and is used to evaluate the general level of the sense of coherence and its three aspects: comprehensibility, manageability and meaningfulness. The results are expressed as the sum of points calculated using the key. The score of <117 points indicates a low level, 117–156 points—a moderate level and >156 points—a high level of the sense of coherence. The reliability of the Polish version for the subscales ranges from 0.68 to 0.78 and amounts to 0.92 for the overall result [17].

The PSQ consists of 27 statements. Separate questionnaires for female and male respondents have been created and three dimensions have been specified, i.e., emotional strain, external stress, and internal stress. The total score indicates the general level of stress experienced by the respondent. The higher the score, the greater the sense of stress. The Cronbach’s alpha, i.e., the internal consistency coefficient, for the three subscales ranges from 0.69 to 0.81 [18]. Raw results were used in the analysis.

### 2.3. Data Analysis

Descriptive statistics were used to compile the characteristics of the sample group. In the statistical analysis, the Shapiro–Wilk test was used to verify the normality of distribution. Due to the result obtained (normal distribution), the statistical analysis was carried out using parametric tests. The findings were compared between two groups of students using the independent sample *t*-tests and the analysis of variance (ANOVA) for three groups with at least five cases in a group. The results of the initial study and the final study were compared using the *t*-test for dependent groups. In the case of the *t*-test, the Bonferroni correction was applied. The relationship between two pairs of variables was calculated using Pearson’s correlation. The statistical tests were verified at the significance level of *p* < 0.05 and *p* < 0.025 in the case of *t*-test results with Bonferroni correction. The calculations were performed using Stastistica 12 StatSoft Poland (StatSoft, Inc., Tulsa, OK, USA). 

## 3. Results

The mean BDI score in the sample group in the initial study (T1) was 8.1 (±6.2) and in the final study (T2)—9.5 (±7.7). In the initial study, the symptoms of mood disorders were found in 28 (25.5%) participants; after two years (T2), the number of students exhibiting symptoms of depression increased to 34 (31%). The mean PSQ score in T1 was 52.0 (±15.5) and in T2—53.7 (±18.2). The mean SOC-29 score in T1 was 136.6 (±20.1). The majority of the students (73; 66%) demonstrated a moderate level of the sense of coherence; a high level of the sense of coherence was found only in 20 (18%) students. After two years of education, the mean SOC-29 score decreased slightly and amounted to 134.7 (±24.6). After this period, an increase in the number of students with low and high levels of the sense of coherence (31 (28%) and 28 (25.5%), respectively) and a decrease in the number of students with a moderate level of the sense of coherence (51; 46%) were recorded.

A statistically significant deterioration in the students’ mood (the BDI scale) was observed in the final study, in particular in the emotional part. On the other hand, no statistically significant difference was recorded between the initial and final results in terms of the perceived stress level of the students (the PSQ scale) and their sense of coherence (SOC-29). These results were not significant after the Bonferroni correction (Table 2).

In T1, no statistically significant difference in the mood between female and male participants was observed. In contrast, in T2, the female students were characterized as having worse moods as compared to the male students. Furthermore, significant mood deterioration after two years of education was found in the female group (*p* = 0.0246). InT1, the mean BDI scores were also statistically significantly higher in the students who declared the willingness to receive psychological help, the students who were not satisfied with the field of study they had chosen and the students who claimed that their studies did not prepare them for work as physiotherapists. After two years (T2), the aforementioned groups of students continued to show significantly worse moods; the statistically significant differences were not recorded only in terms of the preparation for professional work. With the application of Bonferroni correction, statistical significance was observed only in the students who declared the willingness to receive psychological help (in T1and T2) and the students who were not prepared for work as physiotherapists (Table 3).

In both T1and T2, the mean PSQ score of female participants was statistically significantly higher than that of male participants. On the other hand, no statistically significant changes in the perceived stress level in both groups were recorded after two years. InT1, the mean PSQ scores were also significantly higher in the students who declared the willingness to receive psychological help and the students who claimed that their studies did not prepare them for work as physiotherapists. Additionally, after two years (T2), the mean PSQ score was higher in the students who did not work while studying (Table 4).

Male participants were characterized by a statistically significantly higher general sense of coherence as compared to the female participants in both T1and T2. This applies also to the Comprehensibility and Manageability subscales in T1 (*p* = 0.0337; *p* = 0.0008) and all three subscales in T2 (*p* = 0.0128; *p* = 0.0136; *p* = 0.0218). No statistically significant change in SOC-29 scores was recorded after two years. The other data is presented in Table 5.

InT1, lower BDI scores were observed in the group of students who declared that they regularly engaged in physical activity. InT2, the same group continued to show statistically significantly lower BDI scores (in particular in somatic parts), significantly lower PSQ scores (in particular in terms of the emotional strain) and higher SOC-29 scores (in particular in the Meaningfulness subscale) (Table 6).

A statistically significant positive correlation was shown between BDI (in T2) and PSQ (in T2), PSQ (in T1) and BDI (in T2), while a statistically significant negative correlation was observed between BDI (in T2) and SOC-29 (in T2), PSQ (in T2) and SOC-29 (in T2), SOC-29 (in T1) and BDI (in T2).

## 4. Discussion

The preliminary findings published in WORK—A Journal of Prevention Assessment & Rehabilitation (2020) encouraged the authors to continue their research and monitor the emotional state of the students who continued their education during the Master’s degree programme in physiotherapy. The analysis of the results revealed an alarming upward trend in the incidence of mood disorders in this group of students. Despite the fact that the mean BDI scores were not indicative of any symptoms of depression, the percentage of individuals who required psychological/ psychiatric support increased from 25% to 31%. An increase in the number of symptoms of depression can be observed in the studies carried out on physiotherapy students in 2008 and 2014 [5,19,20].

On the other hand, a continuing trend was observed, i.e., despite significant mood deterioration in the evaluated participants, their perceived stress level was low and their level of the sense of coherence was moderate. This may result from effective stress-coping strategies, which have not been studied and should perhaps be included in future research. After two years of education, female physiotherapy students continued to exhibit significantly worse mood, a higher perceived stress level and a lower level of the sense of coherence as compared to the male students. These findings are not surprising for the authors and are consistent with the results obtained by other authors [21,22,23]. It should also be noted that regular physical activity was reported by 65% of female students and 74% of male students at that time point. It demonstrates that in the sample group, male students engaged in physical activity more often. Some numerous factors and theories confirm the differences in the incidence of emotional disorders between genders, from biological factors (e.g., hormonal imbalance), through psychological and social factors, to stress-coping strategies.

However, it is concerning that these results clearly indicate that the mood in this group of female students deteriorates during subsequent years of education.

Taking into consideration other factors covered by the study, it can be concluded that upon finishing their education, the participants who declared the willingness to receive psychological help and expressed dissatisfaction with the field of study they had chosen, the participants who did not work, as well as the participants who claimed that their studies did not prepare them for work as physiotherapists were characterized by worse mood, a higher perceived stress level and a lower level of the sense of coherence. These factors were also mentioned in the initial study carried out on a larger group of students (N = 249), which identified the stress level, the emotional state, and the sense of coherence [13], as well as in the research conducted by other authors [5,22,24].

Declaring the willingness to get psychological help is not equivalent to actually seeking and receiving it. We do not know how many students actually received such help over two years. However, we can presume that as they continued their education at the Department of Physiotherapy, the students’ knowledge of stress and stress-coping strategies increased, which may explain the lower results in this group inT2. Unfortunately, the results also indicate that the level of stress increased in the group of students who did not declare the willingness to receive psychological help, which confirms the trend described in the literature and observed among the students majoring in medical fields, i.e., that seeking help would suggest that their own coping abilities are inadequate [8].

Notably, a multidirectional relationship was revealed, confirmed by the analysis of the correlation between mood disorders, stress, and the sense of coherence. In both the initial study and the final study, carried out after two years of studying, mood disorders were accompanied by a higher perceived stress level and a lower level of the sense of coherence. It was also recorded that a higher perceived stress level and a low level of the sense of coherence during the third year of higher education is correlated with the deterioration of mood after further two years of studying. The relationship between these three parameters has been thoroughly studied [13,25,26]; it is crucial from the perspective of the students themselves and of the preventive measures that should be implemented among the individuals who are at the highest risk of mood deterioration and who decide to continue their education to become physiotherapists.

The analysis of the findings regarding the students’ physical activity once again demonstrates its protective role [10,27]. The mechanism of the effect of exercise on the mood is particularly important; it is based on two types of theories: psychological (“faith in oneself” and the concept of distractors) and biological (e.g., the beta-endorphin theory and the thermogenic theory) [28,29,30]. The students who regularly engaged in recreational physical activity showed considerably better mood and fewer symptoms of depression, as well as a lower perceived stress level (in particular in terms of the emotional strain). They also achieved higher scores on the sense of coherence scale (in particular in the Meaningfulness subscale). It should be noted that these findings pertained to the students both at the first measuring point (during their third year) and after further two years of education. Similar results were obtained by other authors [5,22,31]. Therefore, it is worthwhile to promote regular physical activity among the students of all years, particularly since—as reported by researchers—on average, the first symptoms of depression appear between the ages of 20 and 30 [13]. It is a unique time in a young person’s life—they have to choose their path, often both personal and professional, and confront both themselves and the burdens they notice in the outside, social world.

It is particularly crucial in physiotherapy students because they are expected to serve as models for their patients in the future. The more benefits they derive from regularly engaging in physical activity themselves and the more they are aware of the role it plays in the preservation of good mental health, the more credible they will appear to their patients and the more able they will be to convince the patients that this is a key factor in their recovery.

The sense of coherence is one of the crucial elements that make up the humans’ ability to cope with day-to-day life, also in the areas related to professional activity. This is also confirmed by the findings of the questionnaires completed by the students. The students who had a job were characterized by the highest level of the sense of coherence compared to the unemployed students or the students who worked from time to time. These differences are particularly visible in Table 2, i.e., among the students who were about to finish their education. A high level of the sense of coherence may influence not only the engagement in health-oriented activities, but also the effectiveness on the labour market and it may ultimately translate into professional success, i.e., finding employment [32].

As the findings discussed above clearly show, it is necessary to monitor the emotional state of the students at each stage of their education and to develop or modify the measures undertaken by higher education institutions in order to provide the students with more effective support, perhaps off-campus to avoid stigmatization [8].

### Limitations

The study presented in this article has several limitations. First and foremost, the studies were based on screening tests and did not involve reaching a medical diagnosis. The sample group consisted of the students of one faculty; therefore, it cannot be regarded as representative. On the other hand, the findings are sufficiently interesting to justify the continuation of the studies with the participation of students of all years. In future research, incorporating the measurement of stress biomarkers and the analysis of the stress-coping strategies adopted by the students should be considered.

## 5. Conclusions

The results presented in this article indicate increase of depressive symptoms during the studies in the group of physiotherapy students.

The factors associated with worse mood, a higher perceived stress level and a lower level of the sense of coherence in the study group after two years of studying included: the female gender, the need to receive psychological support, dissatisfaction with the chosen field of study, unemployment, and the sense of not being prepared for work as physiotherapists.

Physical activity can play a protective role in the prevention and treatment of mood disorders. The students who regularly engaged in physical activity exhibited better mood, a lower perceived stress level, and a higher level of coherence than the physically inactive students.

## Figures and Tables

**Table 1:** Characteristics of the study group (N = 110).

Feature	N (%)
Gender	
Female	79 (71.8)
Male	31 (28.2)
Hometown	
Wroclaw	20 (18.2)
Other	90 (81.8)
Regular physical activity (at least twice a week)	
Yes	75 (68.2)
No	34 (30.9)
Financial situation	
Good	32 (29.1)
Satisfactory	72 65.5)
Bad	6 (5.5)
Paid job	
Never	4 (3.6)
From time to time	47 (42.7)
Often	59 (53.6)
Close person/friend	
Yes	102 (92.7)
No	7 (6.4)
Support of psychologist in case of problem	
Yes	73 (65.5)
No	37 (33.6)
Satisfaction with chosen field of study	
Yes	87 (79.1)
No	23 (20.9)
Being prepared for work as a physiotherapist	
Yes	24 (21.8)
No	86 (78.2)
Work as a physiotherapist	
Yes	102 (92.7)
No	7 (6.4)
I don’t know	1 (0.9)
Leave the country after graduation	
Yes	22 (20.0)
No	85 (77.3)
I don’t know	3 (2.7)

**Table 2:** The BDI, PSQ and SOC-29 results in the group of students in the initial and final studies (Student *t*-test for dependent groups).

Parameters	T1			T2		Student *t*-Test	
	N	Mean	SD	Mean	SD	t	*p*
BDI total	110	8.1	6.2	9.5	7.7	2.07	0.0407 *
Emotional part	110	5.7	4.1	6.6	5.4	2.08	0.0395 *
Somatic part	110	2.6	2.7	3.0	2.8	1.43	0.1564
PSQ total	110	52.0	15.6	54.0	18.2	1.05	0.2970
Emotional Strain	110	19.2	6.9	19.7	7.4	0.57	0.5661
External Stress	110	16.2	5.0	16.7	5.6	0.99	0.3230
Internal Stress	110	16.3	5.7	17.1	7.02	1.41	0.1611
SOC-29 total	110	136.6	20.1	134.7	24.6	−1.1	0.2667
Comprehensibility	110	45.9	7.9	44.6	8.9	−1.8	0.0683
Manageability	110	49.6	8.4	48.2	10.1	−1.9	0.0558
Meaningfulness	110	41.2	6.8	41.9	8.2	1.04	0.2995

BDI, Back Depression Inventory; PSQ, Perceived Stress Questionnaire; SOC-29, Sense of Coherence Questionnaire; T1, Initial results; T2, Final results after two years of education; * statistical significance (*p* < 0.05).

**Table 3:** Comparison of the initial and final BDI scores between the selected groups of students (Student *t*-test for independent groups or the ANOVA variance for three groups with at least five cases).

Feature		BDI Total Results							
		T1				T2			
		N	Mean	SD	*p*	N	Mean	SD	*p*
Gender	Female	71	8.5	5.6	0.1149	71	10.3	6.8	0.0334 *
	Male	39	6.9	5.6		39	5.4	6.8	
Hometown	Wrocław	21	7.1	5.4	0.2119	20	9.2	6.7	0.4388
	Other	89	8.3	5.4		90	9.5	6.6	
Financial situation	Good	46	7.0	5.5	0.1212	32	8.9	6.7	0.8888
	Satisfactory	62	8.4	5.5		72	9.8	6.7	
	Bad	2	21.5	5.5		6	9.5	6.7	
Paid job	Never	20	10.2	5.4	0.1605	4	15.3	6.5	0.0639
	From time to time	67	7.2	5.3		47	10.5	6.5	
	Often	23	8.7	5.3		59	8.2	6.5	
Close person/friend	Yes	101	7.8	5.6	0.0580	102	8.9	6.9	0.0703
	No	9	11.2	5.6		8	16.3	6.9	
Support of psychologist	Yes	62	9.2	6.2	0.0180 **	72	8.1	6.8	0.0037 **
	No	48	6.7	5.5		38	12.1	6.8	
Satisfaction with chosen field of study	Yes	100	7.8	5.6	0.0391 *	87	8.7	6.8	0.0303 *
	No	10	11.4	5.6		23	12.1	6.8	
Being prepared for work as a physiotherapist	Yes	60	6.7	5.5	0.0047 **	24	7.9	6.7	0.1331
	No	50	9.8	5.5		86	9.9	6.7	
Work as a physiotherapist	Yes	105	7.9	5.6	0.1436	102	9.3	6.9	0.1620
	No	5	11.0	5.6		8	11.8	6.9	
Leave the country	Yes	30	9.7	5.4	0.1979	22	10.3	6.7	0.2766
	No	58	7.7	5.4		88	9.2	6.7	
	I don’t know	22	6.8	5.4		0	0	0	

BDI, Back Depression Inventory; T1, Initial results; T2, Final results after two years of education; * statistical significance (*p* < 0.05); ** statistical significance after Bonferroni correction (*p* < 0.025).

**Table 4:** The PSQ scores in T1 and T2 in selected groups of students (Student *t*-test for dependent groups or the ANOVA variance for three groups with at least five cases).

Feature		PSQ Total Results							
		T1				T2			
		N	Mean	SD	*p*	N	Mean	SD	*p*
Gender	Female	79	54.1	27.6	0.0111 **	79	55.9	29.0	0.0128 **
	Male	31	46.6	27.6		31	47.1	29.1	
Hometown	Wrocław	21	53.1	27.4	0.1541	20	49.1	28.8	0.0971
	Other	89	51.8	27.4		90	54.4	28.8	
Financial situation	Good	46	49.5	27.5	0.1069	32	50.8	28.8	0.4900
	Satisfactory	62	53.2	27.5		72	54.1	28.8	
	Bad	2	72.5	27.5		6	59.5	28.8	
Paid job	Never	20	57.1	27.3	0.0904	4	69.5	28.5	
	From time to time	67	49.5	27.3		47	56.6	28.5	0.0288 *
	Often	23	55.0	27.3		59	49.9	28.5	
Close person/friend	Yes	101	51.6	27.7	0.1705	102	52.8	29.0	0.1038
	No	9	56.8	27.7		8	61.8	29.0	
Support of psychologist	Yes	62	55.4	27.5	0.0045 **	72	50.2	29.0	0.0074 **
	No	48	47.7	27.5		38	59.6	29.0	
Satisfaction with chosen field of study	Yes	100	51.3	27.7	0.0633	87	52.3	29.1	0.1044
	No	10	59.2	27.7		23	57.7	29.1	
Being prepared for work as a physiotherapist	Yes	60	47.8	27.5	0.0006 **	24	47.8	28.8	0.0407 *
	No	50	57.2	27.5		86	55.0	28.8	
Work as a physiotherapist	Yes	105	51.5	27.7	0.0637	102	53.4	29.1	0.4253
	No	5	62.4	27.7		8	54.6	29.1	
Leave the country	Yes	30	51.4	27.3	0.3593	22	55.0	28.8	0.3238
	No	58	53.8	27.3		88	53.1	28.8	
	I don’t know	22	48.3	27.3		0	0	0	

PSQ, Perceived Stress Questionnaire; T1, Initial results; T2, Final results after two years of education; * statistical significance (*p* < 0.05); ** statistical significance after Bonferroni correction (*p* < 0.025).

**Table 5:** The SOC-29 scores in T1 and T2 of selected groups of students (Student *t*-test for dependent groups or the ANOVA variance for three groups with at least five cases).

Feature		SOC-29 Total Results							
		T1				T2			
		N	Mean	SD	*p*	N	Mean	SD	*p*
Gender	Female	79	133.7	20.2	0.0076 **	79	131.3	23.3	0.0107 **
	Male	31	144.0	17.5		31	143.3	25.6	
Hometown	Wrocław	21	140.1	17.9	0.1868	20	140.9	24.2	0.1064
	Other	89	135.8	20.4		90	133.3	24.5	
Financial situation	Good	46	142.4	18.0	0.0063 **	32	138.9	25.4	0.2796
	Satisfactory	62	132.7	20.6		72	133.8	24.4	
	Bad	2	127.0	14.0		6	122.2	16.1	
Paid job	Never	20	132.4	20.2	0.3842	4	114.5	7.2	
	From time to time	67	136.4	19.6		47	127.3	22.7	0.0111 **
	Often	23	140.9	20.3		59	141.9	24.3	
Close person/friend	Yes	101	137.2	20.1	0.1450	102	135.0	23.5	0.2811
	No	9	129.8	17.9		8	133.3	36.6	
Support of psychologist	Yes	62	133.0	20.5	0.0162 **	72	137.5	24.1	0.0665
	No	48	141.3	18.4		38	129.7	24.8	
Satisfaction with chosen field of study	Yes	100	138.3	19.3	0.0028 **	87	136.7	24.6	0.0438 *
	No	10	120.0	19.8		23	129.2	23.1	
Being prepared for work as a physiotherapist	Yes	60	142.9	18.4	0.0001 **	24	143.3	24.5	0.0267 *
	No	50	129.1	19.3		86	132.3	24.1	
Work as a physiotherapist	Yes	105	137.4	19.9	0.0236 **	102	135.7	24.4	0.0557
	No	5	119.2	14.8		8	121.3	24.1	
Leave the country	Yes	30	133.4	17.8	0.2414	22	134.4	24.5	0.4755
	No	58	136.0	20.2		88	134.5	24.5	
	I don’t know	22	142.7	21.0		0	0	0	

SOC-29, Sense of Coherence Questionnaire; T1, Initial results; T2, Final results after two years of education; * statistical significance (*p* < 0.05); ** statistical significance after Bonferroni correction (*p* < 0.025).

**Table 6:** Comparison of the mean values of the tested parameters in the groups of physically active and inactive students (Student *t*-test for independent groups).

Parameters	Regular Physical Activity	T1				T2			
		N	Mean	SD	*p*	N	Mean	SD	*p*
BDI total	Yes	73	7.4	5.5	0.0467 *	75	8.4	6.8	0.0185 **
	No	37	9.5	5.5		35	11.7	6.8	
Emotional part	Yes	73	5.1	3.9	0.0638	75	5.9	5.6	0.0409 *
	No	37	6.4	4.5		35	7.8	4.9	
Somatic part	Yes	73	2.3	2.6	0.0714	75	2.5	2.7	0.0113 **
	No	37	3.1	2.8		35	3.8	2.9	
PSQ total	Yes	73	51.5	27.6	0.3259	75	51.2	29.0	0.0295 *
	No	37	53.0	27.6		35	58.2	29.0	
Emotional strain	Yes	73	18.6	7.3	0.0862	75	18.1	7.2	0.0009 **
	No	37	20.5	6.0		35	22.7	7.0	
External stress	Yes	73	16.4	5.5	0.2639	75	16.4	5.7	0.2493
	No	37	15.7	4.2		35	17.2	5.3	
Internal stress	Yes	73	16.1	5.8	0.2935	75	16.4	7.1	0.0968
	No	37	16.7	5.7		35	18.3	6.7	
SOC-29 total	Yes	73	137.7	21.6	0.2168	75	137.5	25.4	0.0403 *
	No	37	134.5	16.4		35	128.5	21.8	
Comprehensibility	Yes	73	46.3	8.3	0.1974	75	45.2	9.3	0.1252
	No	37	44.9	7.1		35	43.1	8.0	
Manageability	Yes	73	50.0	8.9	0.2699	75	49.1	10.4	0.1039
	No	37	48.9	7.3		35	46.4	9.2	
Meaningfulness	Yes	73	41.5	7.1	0.2874	75	43.2	8.4	0.0067 **
	No	37	40.7	6.1		35	38.9	6.9	

BDI, Back Depression Inventory; PSQ, Perceived Stress Questionnaire; SOC-29, Sense of Coherence. Questionnaire; T1, Initial results; T2, Final results after two years of education; * statistical significance (*p* < 0.05); ** statistical significance after Bonferroni correction (*p* < 0.025).
